# Association of toll-interacting protein gene polymorphisms with atopic dermatitis

**DOI:** 10.1186/1471-5945-7-3

**Published:** 2007-03-16

**Authors:** Tobias T Schimming, Qumar Parwez, Elisabeth Petrasch-Parwez, Michael Nothnagel, Joerg T Epplen, Sabine Hoffjan

**Affiliations:** 1 Department of Human Genetics, Ruhr-University Bochum, Bochum, Germany; 2 Private medical practice, Gladbeck, Germany; 3 Department of Neuroanatomy and Molecular Brain Research, Ruhr-University Bochum, Bochum, Germany; 4 Institute for Medical Informatics and Statistics (IMIS), University of Kiel, Kiel, Germany

## Abstract

**Background:**

Atopic dermatitis (AD) is a common inflammatory skin disorder, affecting up to 15% of children in industrialized countries. Toll-interacting protein (TOLLIP) is an inhibitory adaptor protein within the toll-like receptor (TLR) pathway, a part of the innate immune system that recognizes structurally conserved molecular patterns of microbial pathogens, leading to an inflammatory immune response.

**Methods:**

In order to detect a possible role of TOLLIP variation in the pathogenesis of AD, we screened the entire coding sequence of the *TOLLIP *gene by SSCP in 50 AD patients. We identified an amino acid exchange in exon 6 (Ala222Ser) and a synonymous variation in exon 4 (Pro139Pro). Subsequently, these two variations and four additional non-coding polymorphisms (-526 C/G, two polymorphisms in intron 1 and one in the 3'UTR) were genotyped in 317 AD patients and 224 healthy controls.

**Results:**

The -526G allele showed borderline association with AD in our cohort (p = 0.012; significance level after correction for multiple testing 0.0102). Haplotype analysis did not yield additional information. Evaluation of mRNA expression by quantitative real-time polymerase chain reaction in six probands with the CC and six with the GG genotype at the -526 C/G locus did not reveal significant differences between genotypes.

**Conclusion:**

Variation in the *TOLLIP *gene may play a role in the pathogenesis of AD. Yet, replication studies in other cohorts and populations are warranted to confirm these association results.

## Background

Atopic dermatitis (AD) is an inflammatory skin disease characterized by pruritus and chronic or relapsing eczematous lesions that commonly presents during early infancy and affects up to 16% of children [1]. AD has a multifactorial background, with genetic predisposition and environmental factors contributing to disease susceptibility [2]. In industrialized countries AD prevalence has increased during the past decades [3], and it has been postulated in the so-called 'hygiene hypothesis' that the lack of contact to microbial products in early infancy might at least in part be responsible for this increase [4]. There is evidence from prospective studies to support an inverse relationship between AD and exposure to endotoxin, a cell membrane component of gram negative bacteria, early day-care attendance and animal exposure [5].

Recognition of microbial products such as endotoxin is mediated by the innate immune system. Toll-like receptors (TLRs) are a family of evolutionarily conserved receptors that recognize pathogen-associated molecular patterns (PAMPs), leading to an inflammatory response by induction of interleukins and other pro-inflammatory proteins [6]. Polymorphisms in *TLR *genes have been implicated in various diseases [7] including AD [8]. Yet, the effect of genetic variation in TLR downstream signalling pathways has not been sufficiently studied yet. Toll-interacting protein (TOLLIP) is an adaptor protein that acts as an inhibitory factor in the TLR-signalling cascade [9-11]. It functions downstream of MyD88 and TIR domain containing adaptor protein (TIRAP) through inhibition of Interleukin-1 receptor associated kinase 1 (IRAK1) [10] and controls the magnitude of inflammatory cytokine production in response to endotoxin [12]. The *TOLLIP *gene is located on chromosome 11p15 and comprises 6 exons encoding a 274 amino acid transcript. The 11p15 region has so far not been reported as a linkage region for AD in the four published genome screens [13]. Yet, association studies are generally supposed to have a greater power to detect common alleles with modest effects on disease susceptibility than linkage studies [14]. Furthermore, dysregulated inhibition in the TLR-signalling cascade may cause a pathologically increased or reduced inflammatory response, and variations in the *ST2 *gene, encoding another inhibitory protein in the TLR pathway, were recently found to be associated with AD [25]. Therefore, we considered the *TOLLIP *gene an interesting candidate gene for AD.

We screened the entire coding region of the *TOLLIP *gene by single strand conformation polymorphism (SSCP) analysis in 50 AD patients in order to identify coding variation that might play a role for AD pathogenesis. Subsequently, the identified polymorphisms were genotyped in 317 AD patients and 224 healthy controls to evaluate a possible association with AD. In order to provide a more complete and valuable assessment of variation in the *TOLLIP *gene, we additionally typed four non coding polymorphisms (located in the promoter and intronic regions as well as in the 3'UTR) that were chosen from the HapMap database [15].

## Methods

### Subjects

317 unrelated patients with atopic dermatitis were recruited by a consultant specialist for AD (Q.P., Gladbeck, Germany), including 193 children and 124 adults. The AD diagnosis was based on the presence of clinical features, including pruritus, eczema with age-dependent differences in location, xerosis and chronic or relapsing dermatitis. In addition, all investigated AD patients had a positive family history for atopic diseases. 224 control samples from adults without known allergies, asthma or AD were collected in the same private practice as the AD patients. We specifically chose to use non-allergic adults as controls because for diseases as frequent as AD, the risk remains very high for asymptomatic children to develop an allergic disease during childhood or even adulthood [16,17]. The control subjects underwent clinical examination in order to exclude symptoms of AD, asthma or allergic rhinitis, had no self-reported allergies or allergic symptoms and no first degree relatives with known allergic diseases. All patient and control subjects were Caucasians of German origin. Informed consent was obtained from all subjects. The study was approved by the Ethics Committee of the Ruhr-University, and the Declaration of Helsinki protocols were followed.

### Preparation of DNA and RNA

DNA was extracted from EDTA anti-coagulated peripheral blood by using a standard salting-out method [18]. Total RNA samples were extracted from EBV-transformed lymphoblastoid cell lines using TriFast reagent (Peqlab) according to the manufacturer's instructions up to the point of phase separation. Afterwards, the aqueous phase was transferred to an RNeasy column (Qiagen, Hilden, Germany) and processed according to the manufacturer's protocol. OD_260 _and OD_280 _were determined and the RNA was stored at -70°C until use.

### Single strand conformation polymorphism analysis

The entire coding region of the *TOLLIP *gene was screened for polymorphisms by polymerase chain reaction (PCR) with consecutive single strand conformation polymorphism (SSCP) analysis. PCR reactions were performed in a total volume of 10 μl, containing 50 ng DNA, 200 mmol of each dNTP, 0.4 U Taq polymerase (Genecraft, Münster, Germany), GC-Buffer (Genecraft, Münster, Germany), and 0.1 μl [^32^P] α-CTP (10 mCi/ml). Thermal cycling was conducted in a thermal cycler (Biometra, Göttingen, Germany). After a denaturation step at 95°C for 5 minutes followed by two initial cycles at 6°C and 3°C above the annealing temperature, 28 cycles with 95°C (30 sec), annealing temperature (30 sec) and 72°C (30 sec) were run. Further details for primers and conditions are given in table 1. For SSCP analysis, 3 μl of the PCR product was mixed with 7 μl of SSCP loading buffer (95% formamide, 20 mM EDTA, 0.05% xylene cyanol and 0.05% bromophenol blue), denatured for 5 minutes at 95°C and thereafter directly cooled on ice. Then 2.5 μl were applied on 6% polyacrylamide gels (38% acrylamide, 2% bisacrylamide) containing 1× TBE buffer (890 mM Tris-borate, 20 mM EDTA, pH 8.3) and 10% glycerine in a SQ3 apparatus (Hoefer, Freiburg, Germany) at 55 W for about 210 minutes. The room temperature was constantly held at 4°C during SSCP analyses.

### DNA sequencing

DNA samples showing mobility shifts on SSCP gels were directly sequenced on an automated capillary DNA sequencer (MegaBace 1000, Amersham Biosciences), using the BigDye cycle sequencing kit (Applied Biosystems, Darmstadt, Germany) as described in the manufacturer's instructions.

### Genotyping

All six polymorphisms in the *TOLLIP *gene were genotyped by restriction enzyme digestion (see Table 1). Patient and control samples were amplified using the same primers and conditions as described above, except for the inclusion of radioactivity. After digestion with the respective enzymes for at least three hours, the fragments were separated on 2% agarose gels in 1× TBE buffer (45 min, 200 V) and visualized by staining with ethidium bromide.

### Measurement of mRNA levels by quantitative real-time PCR

We carried out real-time PCR on an I-Cycler (Bio Rad Laboratories, Hercules, USA). The quantity of PCR products was determined after each round of amplification using the fluorescent dye SYBR Green I (Qiagen), which binds double-stranded DNA. The primer sequences (F: 5'-AGTACGGAGGCGCAGTGG-3'; R: 5'-AGGCGCAGTCGGCAGTAG-3') represent parts of two adjacent exons in order to prevent amplification of potential traces of contaminating genomic DNA. PCR reactions were run in triplicates (95°C for 5 min, 56°C for 30 sec, 95°C for 1 min, 55°C for 30 sec). Fluorescence was recorded at the end of each extension step, and a melting curve was obtained at the end of each run. Quantification of the house-keeping gene glyceraldehyde-3-phosphate dehydrogenase- (GAPDH-) mRNA (primers F: 5'-TGTGTCCGTCGTGGATCTGA-3'; R: 5'-CCTGCTTCACCACCTTCTTGA-3'; product size 76 bp) was used as a control for data normalization. After the PCR, products were analyzed on agarose gels in order to verify band sizes and purity. Expression was assessed by evaluating threshold cycle (C_T_) values. The C_T _values were calculated by the system software (iCycler), and the relative amount of expressed RNA was calculated using Livak 's method [19].

### Statistics

Allele and genotype frequencies were compared between the groups of patients and healthy individuals using a χ^2 ^test. Additionally, we used logistic regression to test for significant associations between AD status and single SNPs alone as well as additional pairwise interactions between SNPs in the *TOLLIP *gene. Significance was assessed by a Wald test on significant deviations of the regression parameters from 0. Correction for multiple testing of SNPs that are in LD with each other was applied according to the method introduced by Li & Ji [20] which improves an approach proposed by Nyholt [21], and consequently single-test p-values < 0.0102 were considered to be significant. Deviations from Hardy-Weinberg equilibrium were evaluated using the FINETTI program. The FamHap software [22] was used for haplotype frequency estimation. 95% confidence intervals for the haplotype frequency estimates were constructed using 10,000 bootstrap samples with replacement (non-parametric bootstrap). We performed power analyses with the Genetic Power Calculator program [23]. Potential binding sites for transcription factors at the -526 C/G locus were evaluated using a transcription factor prediction program [24]. For quantitative real-time PCR, the relative amount of expressed RNA was calculated by using Livak's method [19]. Differences between genotypes were assessed by using the REST 2005 beta V1.9.9 software [25].

## Results

Screening of the coding region of the *TOLLIP *gene revealed two exonic polymorphisms: a synonymous variation in exon 4 (Pro139Pro) and an amino acid substitution in exon 6 (Ala222Ser). Four additional SNPs in non-coding regions (-526 C/G, Intron1a, Intron1b, 3'UTR) were chosen from the HapMap database [15]. Evaluation of these polymorphisms in 317 AD patients and 224 controls showed a modest association of the -526 C/G promoter SNP with AD (table 2). The C allele of the -526G/C promoter polymorphism was significantly more frequent in AD patients than in healthy controls (12.7% *vs*. 7.7%, uncorrected p-value = 0.012). This polymorphism was in strong linkage disequilibrium (LD) with the Intron1a SNP, and the Pro139Pro variation was in moderate LD with the 3'UTR SNP (r^2 ^= 0.64 and 0.20, resp.). Therefore, we felt that Bonferroni correction for independent tests would be overly conservative and applied Li & Ji's multiplicity correction for SNPs that are in LD with each other and, thus, dependent [20]. This approach yielded a significance level of 0.0102 to keep an overall level of 0.05 in both the cases and the control group. Our finding (p = 0.012), therefore, showed borderline significance, being slightly higher than the significance level. Additionally, the Intron1a G allele was more frequent in AD patients (8.8% *vs*. 5.6%) while the 222Ser allele showed an increased frequency in healthy controls (5.4% *vs*. 2.7%). Yet, significance was not evident after correction for multiple testing for both polymorphisms. For the other three variations, no difference in allele or genotype frequencies was found between AD patients and controls. We repeated our analyses within the subgroup of patients with elevated IgE levels and saw the same trend within this subgroup, with the -526G, the Intron1a G allele and the 222Ala allele being more prevalent in cases than in controls (data not shown). Yet, because of the reduction in sample size, the results did not reach statistical significance. All investigated polymorphisms were in Hardy-Weinberg equilibrium in cases and controls. We performed power analyses with the Genetic Power Calculator [23]. Given a multiplicative model with a genotypic relative risk of 2 for heterozygotes and 4 for homozygotes and a D' of 0.9, we would have 96% power to detect a potential effect. Choosing more conservative parameters, with a genotypic relative risk of 1.5/2.25 and D' of 0.8, would yield only 48% power. Haplotype analysis did not yield significant results (table 3). We did not find evidence for a pair wise interaction between SNPs within the *TOLLIP *gene (data not shown).

**Figure 1:**
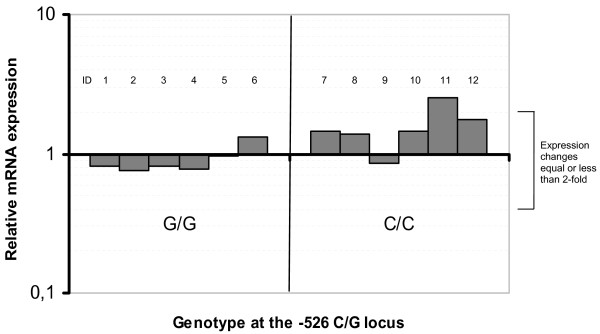
***TOLLIP *mRNA expression in six individuals with the genotype GG (ID numbers 1–6) and six individuals with the genotype CC (ID numbers 7–12) at the -526 C/G locus**. Total RNA samples were extracted from EBV-transformed lymphoblastoid cell lines and mRNA levels measured by quantitative real-time PCR. For each individual, mRNA expression is shown relative to the mean expression of the six individuals with the opposite genotype. The range of expression changes is less than 2-fold for each individual except ID11. No significant differences in *TOLLIP *mRNA expression between genotypes were obvious.

Because of the observed borderline association of the -526 C/G polymorphism with AD, we searched for potential binding sites for transcription factors at this locus using a transcription factor prediction program [24]. Differential binding of two transcription factors was predicted for the two different alleles at this locus. While the G allele gave a putative binding site for the AP2-γ transcription factor, the C allele showed a putative binding site for E2F. The mRNA expression in B-lymphocyte cultures from six probands with the C/C genotype and six probands with the G/G genotype did not show any differences in expression between the genotypes (figure 1).

## Discussion

The innate immune response initiated by TLRs is an important mechanism in defense against pathogenic microorganisms, and variations in *TLR *genes have been implicated in the pathogenesis of allergic as well as autoimmune diseases [7]. Several studies have pointed out that the innate immune system plays an important role in AD pathogenesis. For example, epidemiological studies showed an inverse relationship between AD and exposure to endotoxin (lipopolysaccharide, LPS), early day-care attendance and animal exposure [5], suggesting that lack of contact to microbial products might increase risk for AD. Further, TLRs were found to be up-regulated in circulating monocytes from AD patients [26]. A polymorphism in the *TLR2 *gene was associated with a severe AD phenotype [8]. Additionally, variations in the *CD14 *gene, encoding part of the cell membrane receptor for LPS, as well as in the *CARD15 *gene, encoding an intracellular LPS receptor, have shown associations with AD [27,28]. Thus, genes in the downstream pathway of TLR signalling also constitute reasonable candidate genes for this frequent skin disease. We demonstrate here first evidence for an association of AD with variation in the *TOLLIP *gene, encoding a protein with an inhibitory function in the TLR signalling pathway.

Screening of the coding region of the *TOLLIP *gene by SSCP identified two coding variations: Pro139Pro and Ala222Ser. The rate of detection of nucleotide variants by SSCP varies between 80% and close to 100% under optimized conditions [29]. Thus, it is possible that rare variations might have been missed with this approach. Yet, for multifactorial diseases like AD, common instead of rare variations have been suggested to play a role for pathogenesis [30]. Therefore, we chose to additionally type four frequent SNPs in non-coding regions from the HapMap database. We found a modest association of a promoter polymorphism (-526 C/G) in the *TOLLIP *gene with AD. Since Bonferroni correction has been controversial for genetic association studies because it might be overly conservative [31], we chose to use a method for multiplicity correction when SNPs in linkage disequilibrium (LD) are tested that was introduced by Li & Ji [20]. Our observed p-value of 0.012 for the -526 C/G promoter SNP was borderline significant with respect to Li & Ji's method (0.0102). We therefore believe that it might represent a true association. Haplotype analysis did not yield additional information.

Interestingly, functional SNPs in the distal promoter of the *ST2 *gene were recently found to be associated with AD in the Japanese population [32]. Both TOLLIP and ST2 exert inhibitory functions in the TLR cascade [9,33], and for the *TOLLIP *gene we also saw the most significant although borderline association with the promoter SNP. Therefore, we decided to further explore the role of the -526 C/G variation for AD. Investigation of potential binding sites for transcription factors at the -526 C/G locus using a transcription factor prediction program predicted differential binding of two transcription factors (AP2-γ and E2F) for the two different alleles at this locus. Interestingly, E2F is an important factor in the control of skin proliferation [34], and the AP2 family appears to regulate the expression of genes required for the development of ectodermal tissues, including skin [35]. In order to detect a possible direct influence of the *TOLLIP *-526C/G polymorphism on mRNA expression, we performed quantitative real-time PCR for measurement of mRNA amounts in lymphoid cell cultures from six probands with the C/C and six with the G/G genotype at this locus. Yet, we were unable to find differences in mRNA expression with this method. There are several explanations for this finding. First, a variation that is more distal to the promoter and in LD with the -526 C/G polymorphism or some promoter haplotypes might be the true disease-associated variation. In fact, the recently identified functional SNPs in the *ST2 *promoter are also located very distal from the transcription start (-26999 and -27639, resp. [32]). Second, measuring mRNA expression *via *quantitative real-time PCR may not represent the most sensitive method to detect moderate differences in promoter function. Third, expression studies in keratinocytes instead of blood B-lymphocytes would be more informative for a chronic skin disease such AD. Finally, since our association results are somewhat borderline, there may indeed be no functional significance of the analyzed promoter SNP. Yet, functional studies including electro-mobility shift assays (EMSA) are needed to further evaluate the functional relevance of the -526 C/G promoter polymorphism in the *TOLLIP *gene.

We cannot exclude the possibility that the association result we found for the *TOLLIP *promoter polymorphism may be caused by another SNP in *TOLLIP *or a neighbouring gene that is in LD with this variation. For example, the intron 1a SNP that is in LD with the -526C/T polymorphism and the Ala222Ser variation in exon 6 also showed marginal evidence for an association with AD. Further, we are aware of the fact that genetic association studies bear the risk of false-positive results caused by hidden population substructures. Yet, all our patients and controls were of German origin and recruited in North Rhine Westfalia. Even if some of the patient families and controls might originate from neighbouring European countries, a recent study demonstrated that linkage disequilibrium patterns are conserved across European samples for most gene regions [36]. Thus, we consider the risk that our results are caused by hidden population substructures to be marginal, at most. Yet, population stratification could in principle contribute to the borderline significant results we observed in our association study. Finally, the relatively small size of our sample bears the risk of small power, potentially causing false-negative association results. Yet, power analyses indicated that considering a multiplicative model with a genotypic relative risk of 2 for heterozygotes and 4 for homozygotes and a D' of 0.9, we would have a 96% power to detect a potential effect of a chosen marker. Choosing more conservative parameters, with a genotypic relative risk of 1.5/2.25 and D' of 0.8, would yield only 48% power. On the other hand, the cohort we present here has been thoroughly recruited by a single physician, so that it constitutes a highly controlled sample with exclusion of many potential confounders. In any case, the borderline evidence for association of *TOLLIP *variation with AD that we present in this study clearly needs to be replicated in additional studies and populations in order to rule out false positive results.

## Conclusion

We present first evidence for an association of *TOLLIP *variation with atopic dermatitis. Our results, although borderline, support the concept that genetic variation in the TLR system may play an important role in the pathogenesis of AD. In combination with recently published association findings for the *ST2 *gene [32], we suggest that dysregulation of inhibition in the TLR pathway might be of special importance. Yet, the exact mechanism by which variation in negative regulators of TLR signalling may influence AD pathogenesis still needs to be explored. Targeting TLRs has already been discussed as a novel therapeutic option for allergic diseases [37,38]. Thus, better understanding of the molecular pathogenesis of AD could eventually lead to new options in prevention and treatment of this frequent skin disease.

## List of abbreviations used

AD atopic dermatitis

LD linkage disequilibrium

PAMP pathogen-associated molecular pattern

PCR polymerase chain reaction

SNP single nucleotid polymorphism

SSCP single strand conformation polymorphism

TLR Toll-like receptor

TOLLIP Toll-interacting protein

## Competing interests

The author(s) declare that they have no competing interests.

## Authors' contributions

TS performed the experiments and drafted the manuscript. QP and EPP recruited the patients as well as controls and collected clinical data. MN performed the statistical analyses. JTE participated in the design and coordination of the study. SH was in charge of the design and coordination of the study as well as the statistical analyses and finalised the manuscript. All authors read and approved the final manuscript.

## Pre-publication history

The pre-publication history for this paper can be accessed here:



## Figures and Tables

**Table 1:** Primer sequences, PCR conditions and restriction enzymes used for mutation analysis and genotyping of selected polymorphisms in the *TOLLIP *gene.

Amplicon	Rs number	Amino acid	Primer	Annealing temperature	MgCl_2 _(mmol/l)	Restriction enzyme
*TOLLIP*-526 C/G	5743854	-	**F**: GGGTGACCTTCGGACGTG **R**: CATCTTTAGCCCTGTGAAACAG	57°C	2	Hin6I
*TOLLIP *exon 1*	-	-	**F**: GACAACTGTCTCCGTCGCA **R**: TGGGTCAGGGCTACTGCT	55°C	2	-
*TOLLIP *intron1a	5743867	-	**F**: ACTCTCCCAGCACCTGACGCTTAAG **R**: ACCAGCGCTACCAACTGTGGCGAT	61°C	4	MboI
*TOLLIP *intron1b	11042484	-	**F**: GACCCAGCCGTCTGCAGGG **R**: TCCATGGCACAGGCAGGTACG	62°C	2	Hin6I
*TOLLIP *exon 2*	-	-	**F**: ACCCTGACTCACTGGCCTC **R**: TTTCAACATCACACAGGTTTCA	58°C	2	-
*TOLLIP *exon 3*	-	-	**F**: GTTGGCACCTCCTTTTCCTGAC **R**: CAGGTGTGTCCCCACGGC	66°C	2	-
*TOLLIP *exon 4	3750920	Pro139Pro	**F**: GGGCCTCTCCCTCTTGTC **R**: TCAGAACGTGAGCCCTGC	60°C	3	MspI
*TOLLIP *exon 5*	-	-	**F**: GGTTCTGATGGGTGGATGTTCC **R**: ACCTGCCCTAGGCCCTGG	66°C	2	-
*TOLLIP *exon 6	5744015	Ala222Ser	**F**: TTCCGACGTTTTTACCAAACTTGAT **R**: GCGTGTCCAAAGAGCGGG	63°C	2	MwoI
*TOLLIP *3'UTR	3750919	-	**F**: AGCCTGACTCTCACACTCAGCA **R**: GGGAGCAAAACCTTGATACCAA	58°C	3	Alw26I

F = forward primer, R = reverse primer,

*Amplicons were only amplified for SSCP analysis; no coding variation was discovered with this method

**Table 2:** Genotype and allele frequencies of *TOLLIP *polymorphisms in AD patients and healthy controls*.

Polymorphism	Genotype	Allele	AD	Controls	p-value
-526 C/G	C/C		240 (76.9%)	181(84.6%)	
	C/G		65 (20.8%)	33 (15.4%)	
	G/G		7 (2.2%)	0 (0%)	
	n		312	214	0.08
		C	545 (87.3%)	395 (92.3%)	
		G	79 (**12.7%**)	33 (**7.7%**)	
		n	624	428	**0.012**

Intron1a	A/A		265 (83.6 %)	199 (88.8%)	
	A/G		48 (15.1%)	25 (11.2%)	
	G/G		4 (1.3%)	0 (0%)	
	n		317	224	0.16
		A	578 (91.2%)	423 (94.4%)	
		G	56 (8.8%)	25 (5.6%)	
		n	634	448	0.045

Intron1b	A/A		2 (0.6%)	3 (1.3%)	
	A/G		54 (17.3%)	39 (17.5%)	
	G/G		256 (82.1%)	181 (81.2%)	
	n		312	223	0.903
		A	58 (9.3%)	45 (10%)	
		G	566 (90.7%)	401 (90%)	
		n	624	446	0.66

Pro139Pro	C/C		92 (30.4%)	64 (30.0%)	
	C/T		147 (48.5%)	113 (53.1%)	
	T/T		64 (21.1%)	36 (16.9%)	
	n		303	213	0.43
		C	331 (54.6%)	241 (56.6%)	
		T	275 (45.4%)	185 (43.4%)	
		n	606	426	0.52

Ala222Ser	Ala/Ala		293 (94.5%)	198 (89.6%)	
	Ala/Ser		17 (5.5%)	22 (9.6%)	
	Ser/Ser		0 (0%)	1 (0.5%)	
	n		310	221	0.049
		Ala	603 (97.3%)	418 (94.6%)	
		Ser	17 (2.7%)	24 (5.4%)	
		n	620	442	0.02

3'UTR	G/G		193 (61.5%)	133 (60.5%)	
	A/G		105 (33.4%)	76 (34.6%)	
	A/A		16 (5.1%)	11 (5.0%)	
	n		314	220	0.965
		G	491 (78.2%)	342 (77.7%)	
		A	137 (21.8%)	98 (22.3%)	
		n	628	440	0.86

*According to the method of multiplicity correction for SNPs in LD that was introduced by Li & Ji [20], we considered p-values < 0.0102 as significant.

**Table 3:** *TOLLIP *haplotype frequencies in AD patients and controls.

Haplotype	Frequency in AD patients (n = 312)	95%-CI*	Frequency in controls (n = 222)	95%-CI*
C-A-G-T-G-G	0.437	0.396–0.477	0.421	0.376–0.466
C-A-G-C-G-A	0.202	0.169–0.236	0.205	0.169–0.245
C-A-G-C-G-G	0.106	0.081–0.133	0.138	0.104–0.175
C-A-A-C-G-G	0.091	0.069–0.114	0.090	0.063–0.119
G-G-G-C-G-G	0.083	0.061–0.107	0.055	0.034–0.076
G-A-G-C-G-G	0.031	0.016–0.046	0.023	0.009–0.039
C-A-G-C-T-G	0.026	0.014–0.039	0.041	0.022–0.061

*95% confidence interval for the haplotype frequency estimate using 10,000 bootstrap samples with replacement (non-parametric bootstrap).
